# Supplementary material for: Modeling microbiome-trait associations with taxonomy-adaptive neural networks
Source: Microbiome. 2025 Mar 29;13:87. doi: 10.1186/s40168-025-02080-3 (PMC11954268; doi:10.1186/s40168-025-02080-3)
Supplement: Supplementary file 2 — Supplementary Material 1. [file 40168_2025_2080_MOESM1_ESM.pdf]

Supplement to "Modeling microbiome-trait associations with taxonomy-adaptive neural networks"

Yifan Jiang[1], Matthew Aton[2], Qiyun Zhu[2,*], and Yang Young Lu*[1,*]

[1]Cheriton School of Computer Science, University of Waterloo, Waterloo, Ontario, Canada
[2]School of Life Sciences, Arizona State University, Tempe, Arizona, USA

S1 Dataset details

MIOSTONE used seven publicly available microbiome datasets with varying sample sizes and feature dimensionality, with details listed in Tab. S1 and Fig. S1.

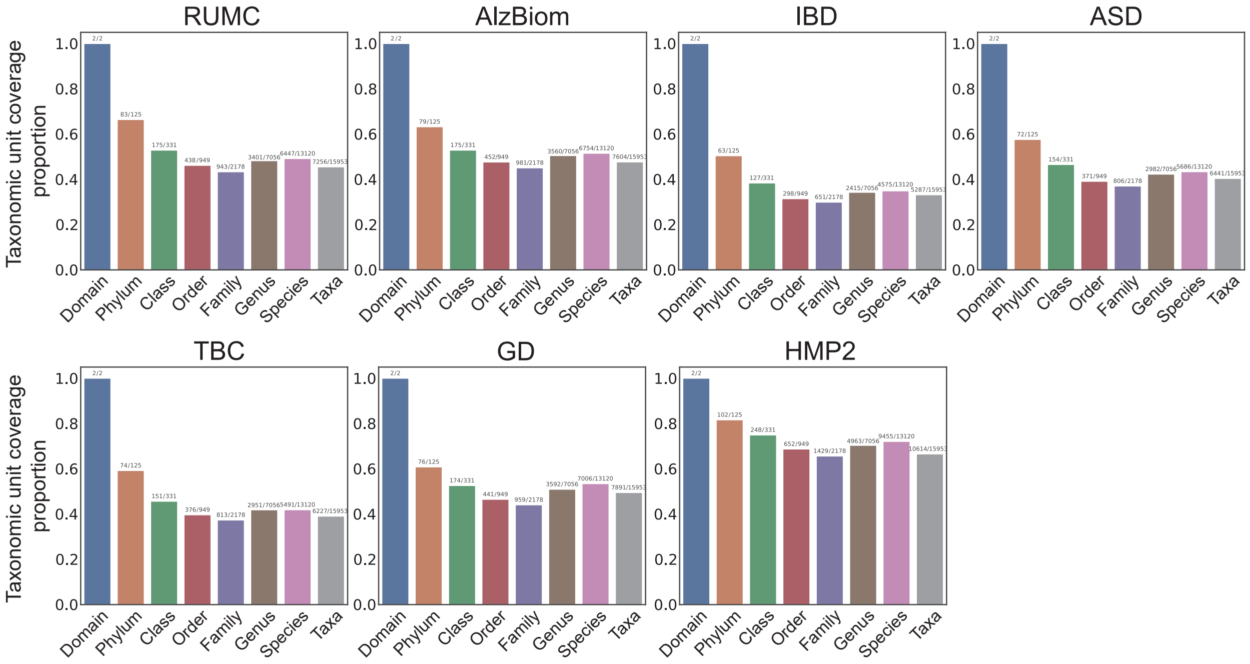

Figure S1: **The microbiome datasets exhibit varying microbial taxa sizes and encompass different proportions of taxonomic levels.**

Additionally, we created two simulated datasets using the microbiome data simulator MIDASim [He et al., 2024]. MIDASim generates simulated data by leveraging a template microbiome dataset and preserving its correlation structure to ensure similarity. Specifically, we employed the parametric mode of MIDASim, utilizing a generalized gamma distribution to model the relative abundances of microbiome data. This approach is tailored for simulation studies that involve modifying the log-mean relative abundance. Since MIDASim is not designed to generate simulated samples

*Correspondence: yanglu@uwaterloo.ca

with labels, we selected a real dataset with positive and negative labels. We then simulated samples separately for each label (positive and negative) before combining them into a unified simulated dataset. The real dataset we chose is the IBD dataset [Gonzalez et al., 2022] studied the relationship between the gut microbiome and two main subtypes of inflammatory bowel disease (IBD): Crohn's disease (CD) and ulcerative colitis (UC). It includes 108 CD and 66 UC samples, with profiles containing $p = 5287$ taxa.

We simulated two distinct datasets from the IBD dataset. We estimated separate location parameters for each of the two labels from the IBD dataset, denoted as $\mu_+ \in \mathbb{R}^{5287}$ and $\mu_- \in \mathbb{R}^{5287}$. After that, we varied the location parameters to represent different levels of difficulty in distinguishing between labels, as follows:

- Setting 1: $(2 * \mu_+)$ and $(2 * \mu_-)$.
- Setting 2: Randomly select 10% of taxa with non-zero abundance and increase their values by 10%.

For each of these two simulated datasets, we generated the same number of positive and negative samples, matching the distribution of the IBD dataset.

Lastly, given that both the two simulated and seven real datasets have small sample sizes and high feature dimensionality, we included the third simulated dataset with a small sample size and low feature dimensionality to evaluate MIOSTONE's performance in an atypical setting. Specifically, we employed the simulated dataset generated by Zhai et al. [2024]. The dataset comprises 48 genus aggregated from $2,964$. We subsampled 50 samples, comprising 32 positive and 18 negative samples, respectively.

S2 Benchmark details

We evaluate the performance of MIOSTONE in comparison to nine baseline methods, divided into two categories: tree-agnostic methods and tree-aware methods. The former category comprises random forest (RF), support vector machine (SVM) with a linear kernel, XGBoost [Chen and Guestrin, 2016], and multi-layer perceptron (MLP), while the latter includes, DeepBiome [Zhai et al., 2024], Ph-CNN [Fioravanti et al., 2018], PopPhy-CNN [Reiman et al., 2020], TaxoNN [Sharma et al., 2020], and MDeep [Wang et al., 2021]. DeepBiome [Zhai et al., 2024] constructs its neural network architecture based on the phylogenetic structure, where each hidden layer corresponds to a specific phylogenetic level (e.g., family, order, class, and phylum). The model is trained with a phylogenetic regularization technique using weight decay to prevent overfitting and improve generalization. Ph-CNN [Fioravanti et al., 2018] is based on a novel Phylo-Conv layer, which combines a convolutional operation with a neighbors detection algorithm. The network consists of a stack of Phylo-Conv layers, which are then flattened and followed by a fully connected (dense) layer, culminating in a final classification layer. PopPhy-CNN [Reiman et al., 2020] is a novel convolutional neural network designed to leverage the phylogenetic structure of microbial taxa for host phenotype prediction. The network processes a 2D matrix input, where the rows represent the phylogenetic tree and the columns contain the relative abundance of microbial taxa in a metagenomic sample. TaxoNN [Sharma et al., 2020] stratifies input taxa into different clusters based on their phylum information. The network comprises an ensemble of convolutional neural networks, each operating on a stratified cluster of taxa that share the same phylum. This approach is based on the rationale that taxa within the same phylum exhibit similarities and potential correlations, which can enhance predictive performance. MDeep [Wang et al., 2021] designs convolutional layers to mimic taxonomic ranks with multiple convolutional filters on each convolutional layer to capture the phylogenetic correlation among microbial species in a local receptive field and maintain the correlation structure across different convolutional layers via feature mapping.

We used the Scikit-learn implementation [Pedregosa et al., 2011] with default settings for the RF and SVM models. Specifically, the random forest classifier is trained with 100 trees, without a maximum tree depth constraint, and a minimum of 2 samples required to split an internal node. The support vector classifier is trained with a linear kernel, using L2 regularization with a coefficient of 1.0. The XGBoost classifier employs gradient boosting with decision trees, using a learning rate of 0.3, a maximum depth of 6, and 100 boosting rounds. For the tree-aware models, we used the recommended implementation settings. The MLP model is configured with a pyramid-shaped architecture with one hidden layer of size half of the input dimensionality. The DeepBiome model incorporates phylogenetic tree-based weight decay, utilizing Xavier uniform initialization without batch normalization or dropout. The Ph-CNN

model consists of two 1D-CNN layers with 4 neighbors and 16 filters each, followed by a fully connected layer with 64 units and a dropout rate of 0.25. The PopPhy-CNN model features a 2D-CNN layer with 32 filters (kernel size: 3×10), followed by a fully connected layer with 512 units and a dropout rate of 0.3. We followed its built-in preprocessing pipeline, which involved log-scaling the normalized relative abundance of each taxon followed by subsequent min-max normalization. The TaxoNN model includes a 1D-CNN layer with 32 filters, another with 64 filters (both with a kernel size of 5), and a fully connected layer with 100 units. We utilized the recommended settings, *i.e.,* clustering the taxa based on their phylum information and ordering them according to Spearman correlation, as reported to yield optimal performance. The MDeep model comprises two 1D-CNN layers with 64 filters each and one layer with 32 filters, all with a kernel size of 8, a stride of 4, and a dropout rate of 0.5. All tree-aware models and MLP are trained for 200 epochs with a batch size of 512 to ensure convergence. During training, we used the AdamW optimizer with a learning rate of 0.001 and applied a cosine annealing scheduler. For a fair comparison, all models were trained and tested in the same environment: AMD EPYC 7302 16-Core Processor, NVIDIA RTX A6000 GPU, with 32GB DDR4 RAM.

S3 Detailed experiments

S3.1 Supervied learning

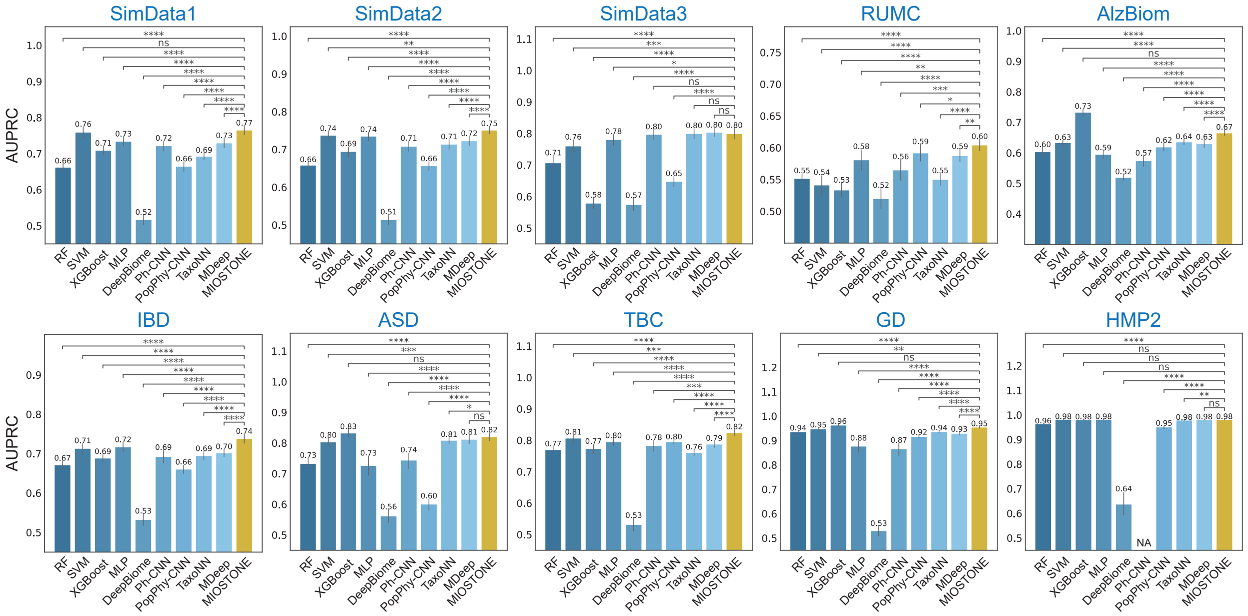

Figure S2: **Performance of MIOSTONE in host's disease status prediction in terms of AUROC.** The evaluation was performed on three simulated and seven real microbiome datasets. MIOSTONE is compared against nine baseline methods, divided into two categories: tree-agnostic methods and tree-aware methods. Each model was trained by times using different train-test splits, and reported by the average performance along with 95% confidence intervals. The models' performances are measured by the Area Under the Receiver Operating Characteristic Curve (AUROC) For scientific rigor, the performance comparison between MIOSTONE and any other baseline method is quantified using one-tailed two-sample t-tests to calculate p-values: $****$ p-value ≤ 0.0001; $***$p-value ≤ 0.001; $**$ p-value ≤ 0.01; $*$: p-value ≤ 0.05; ns : p-value > 0.05.

S3.2 Transfer learning

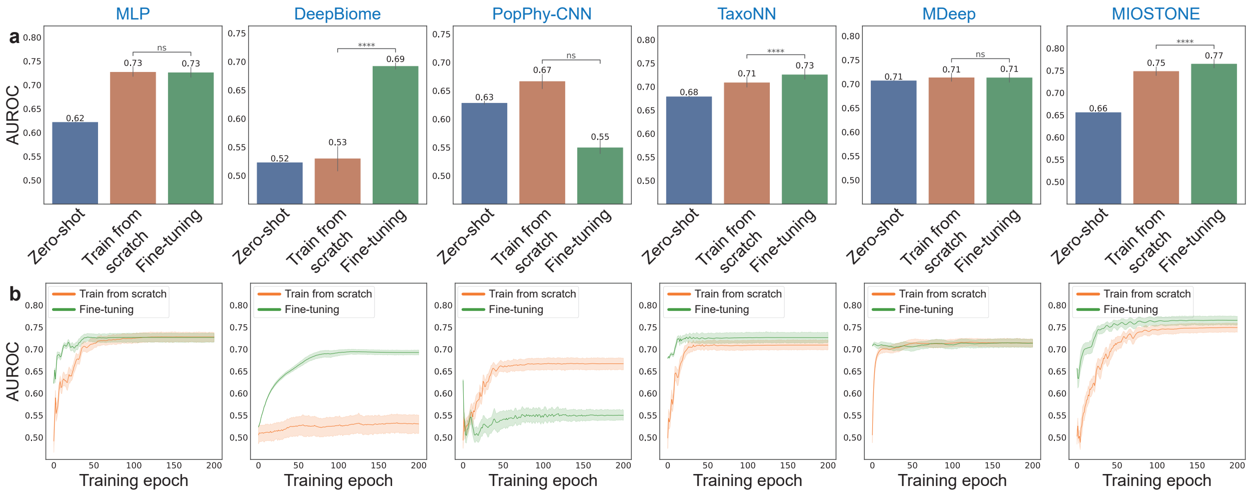

Figure S3: **Performance of MIOSTONE in transferring knowledge from pre-trained models in terms of AUROC.** (**a**) A model on the large HMP2 dataset is pre-trained and then employed for the smaller IBD dataset in three settings: direct prediction on IBD (*i.e.,* zero-shot), fine-tuning on IBD, and training IBD from scratch. Only tree-aware methods and MLP are included in the comparison, as most tree-agnostic methods are not well-suited for fine-tuning. Among the tree-aware methods, Ph-CNN is excluded because it is not scalable for processing the large HMP2 dataset. The prediction is conducted across three settings 20 times with varied train-test splits, and reported by the average performance assessed by AUROC, along with 95% confidence intervals. For scientific rigor, the performance between fine-tuning and training from scratch is quantified using one-tailed two-sample t-tests to calculate p-values. (**b**) The training dynamics of various models were evaluated by comparing fine-tuning with training from scratch, analyzing AUROC on test splits across different training epochs. MIOSTONE's fine-tuning achieved better performance than training from scratch, requiring fewer training epochs.

S3.3 Representation learning

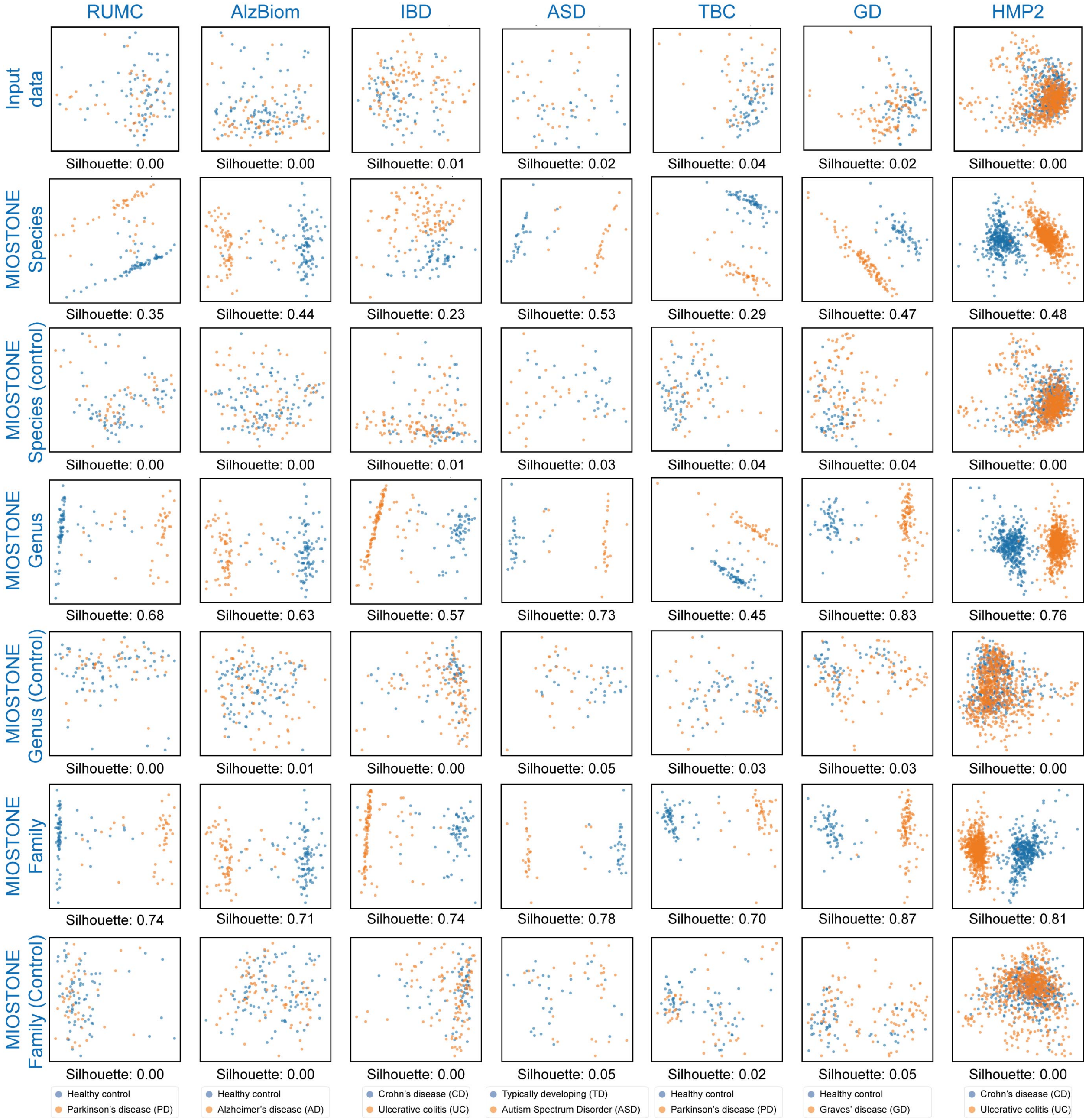

Figure S4: **A sanity check of MIOSTONE's internal neuron representations.** We investigated whether MIOSTONE's internal neuron representations depend on the training data. If the representations were independent of the data and solely reliant on the model's taxonomy-encoding architecture, it would be unreasonable and unreliable to draw convincing conclusions from the model. As a sanity check, we used an untrained MIOSTONE model to project the internal neuron representations of samples onto a two-dimensional Principal Component space and evaluated their ability to distinguish between different disease subtypes, comparing the results to those of the trained MIOSTONE model. The untrained model exhibited no separation between disease subtypes, confirming that the model's internal representations are data-dependent and capture disease-specific signatures during training.

S3.4 Model interpretation

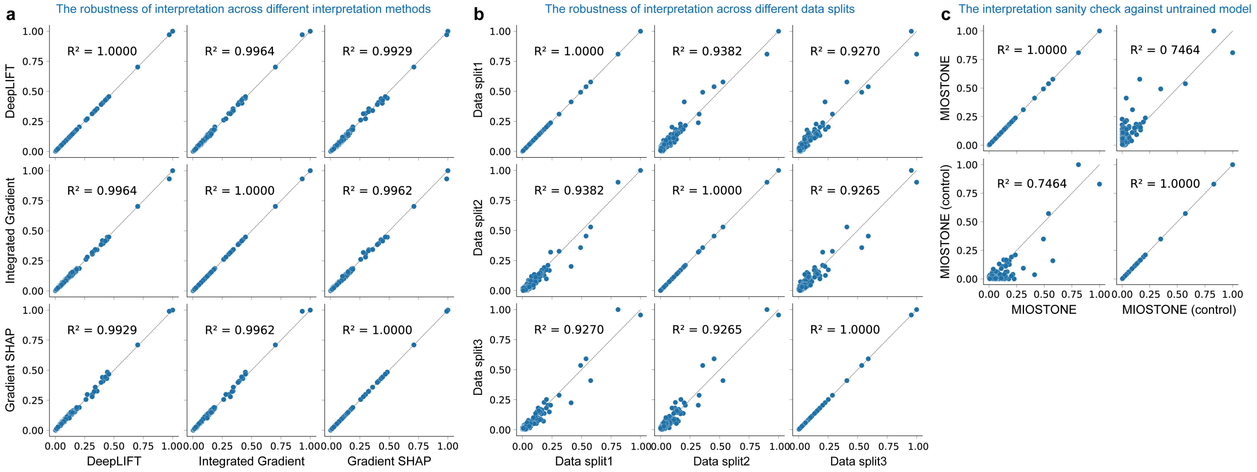

Figure S5: **MIOSTONE is robust in discovering microbiome-disease associations using feature attribution methods.** Feature attribution methods are used to interpret the MIOSTONE model and quantify the relationships between microbiome taxa and disease traits. (**a**) Three mainstream feature attribution methods–DeepLIFT, Integrated Gradients, and SHAP–demonstrate strong consistency in quantifying key microbiome-disease associations derived from the MIOSTONE model. Therefore, the consistency of the methods makes it sufficient to present only the DeepLIFT results. (**b**) DeepLIFT demonstrates strong consistency in quantifying key microbiome-disease associations across different data splits. (**c**) A sanity check of MIOSTONE's discovering microbiome-disease associations using feature attribution methods. We investigated whether the microbiome-disease associations reported by feature attribution methods depend on the training data. As a sanity check, we used an untrained MIOSTONE model and evaluated the consistency between the microbiome-disease associations it reported and those from a trained model. The low consistency suggests that the microbiome-disease associations reported by feature attribution methods are data-dependent.

S3.5 Control studies

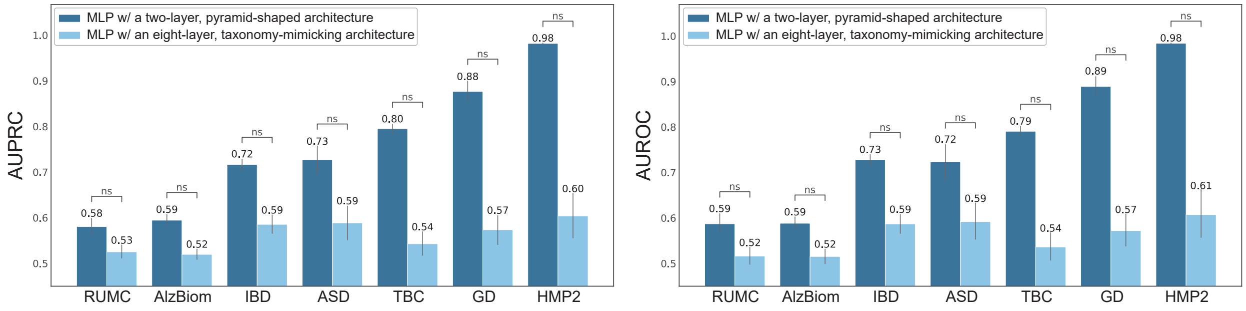

Figure S6: **An MLP with a taxonomy-mimicking architecture does not enhance prediction accuracy.** The MLP model used as baseline is configured with a pyramid-shaped architecture with one hidden layer of size half of the input dimensionality. Alternatively, we designed an MLP model that mirrors the taxonomy architecture, with each hidden layer corresponding to a specific taxonomic level, maintaining the same number of layers and neurons. The alternative MLP design significantly underperforms in prediction, as evidenced by lower AUPRC and AUROC scores. This suggests that taxonomy alone does not account for the superior predictive performance, as the increased number of parameters introduces additional challenges during training.

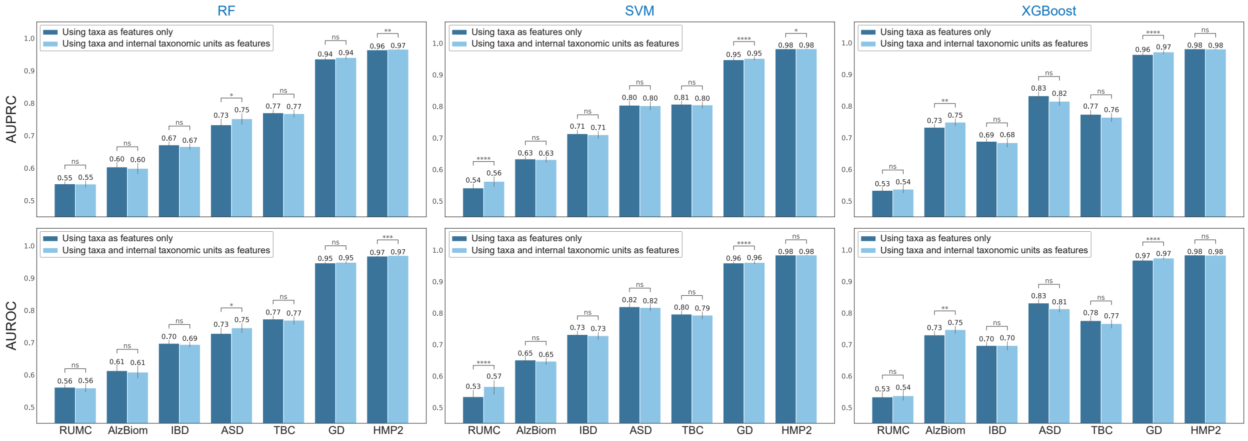

Figure S7: **Flattening the internal taxonomic units as additional features does not effectively exploit the taxonomy.** The feature value of each internal taxonomic unit is computed as the average of the values across all taxa within that specific taxonomic unit. Each tree-agnostic model takes as input the concatenated features from both the taxa and the internal taxonomic units. Treating the internal taxonomic units as additional features results in marginal or even worse predictive performance, as measured by both AUPRC and AUROC.

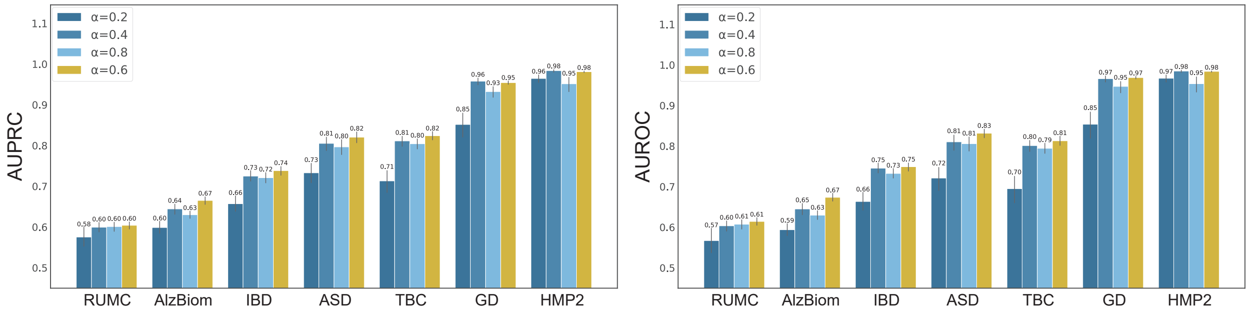

Figure S8: **MIOSTONE demonstrates robustness in selecting the hyperparameter that controls taxonomy-dependent representation dimensionality.**

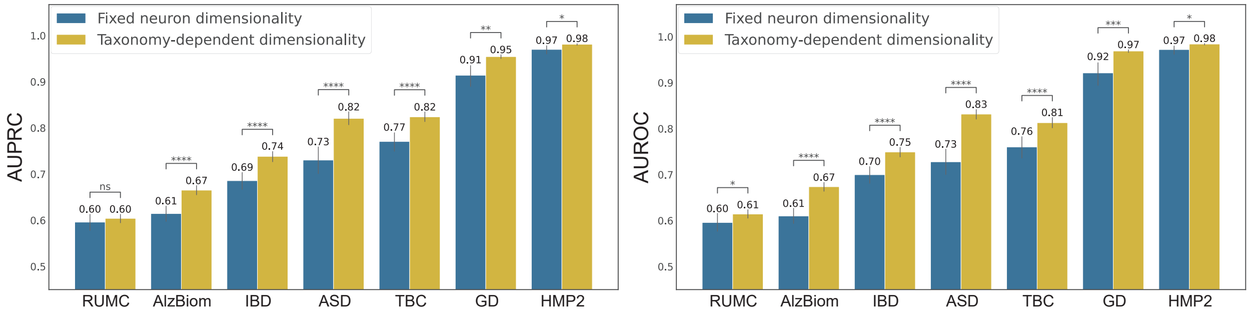

Figure S9: **MIOSTONE demonstrates superior performance in using taxonomy-dependent representation dimensionality.** MIOSTONE's assigning larger taxonomic groups with greater representation dimensionality can aid in capturing more complex biological patterns to predict traits, compared to using fixed representation dimensionality.

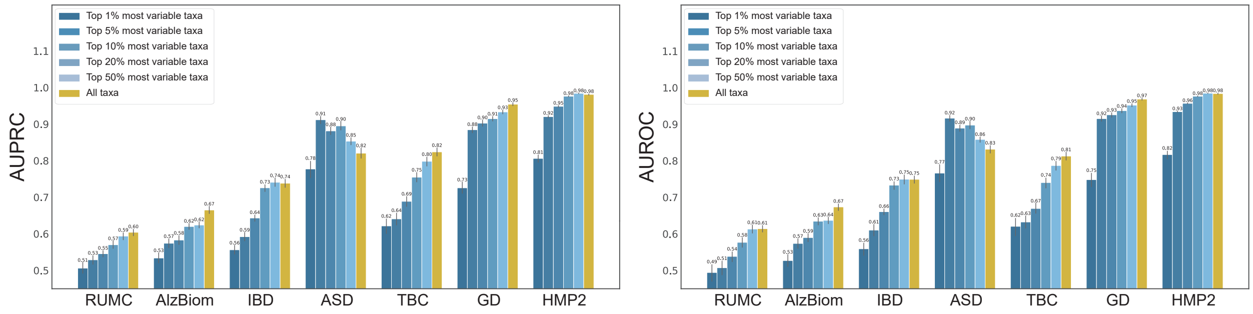

Figure S10: **The curse of dimensionality cannot simply be mitigated using feature selection.** MIOSTONE trained with all microbiome features, either outperforms or matches the performance of the model trained with a subset of highly variable taxa across most datasets.

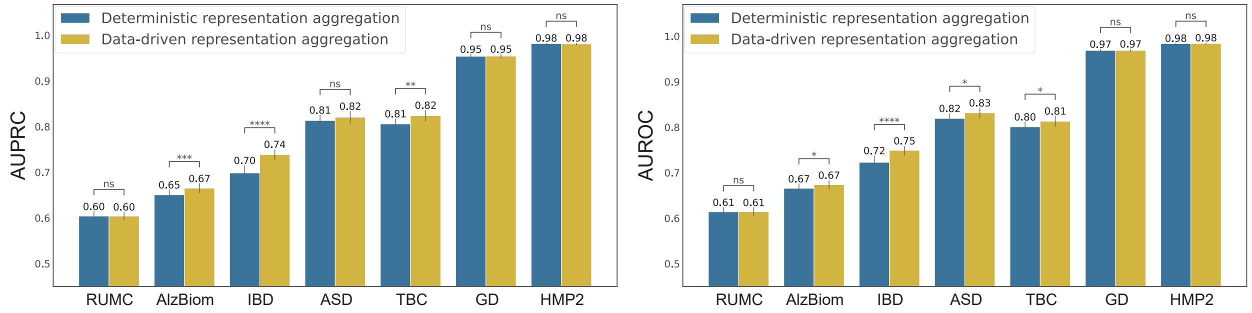

Figure S11: **MIOSTONE demonstrates superior performance in using data-driven aggregation of neuron representations.** MIOSTONE's data-driven aggregation of neuron representations either outperforms or matches the performance of the deterministic selection of nonlinear representations across most datasets.

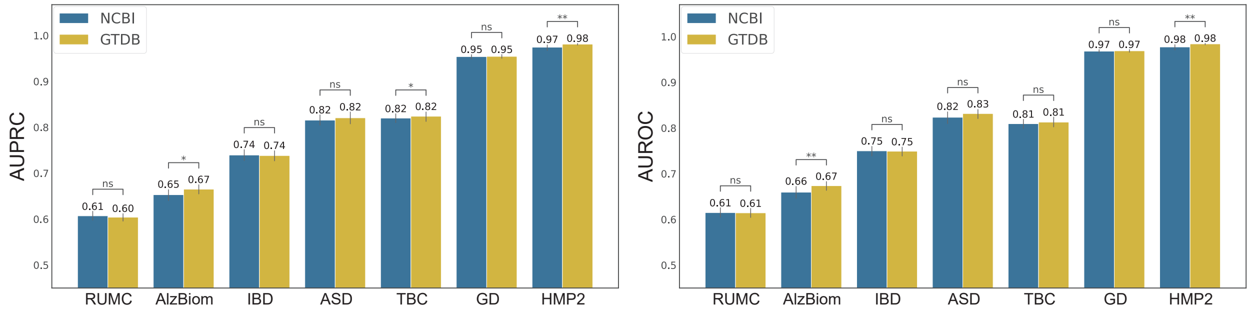

Figure S12: **MIOSTONE demonstrates robustness across various taxonomic trees.** Two variations of MIOSTONE utilizing taxonomies from GTDB and NCBI respectively, demonstrate comparable predictive performance across seven real microbiome datasets.

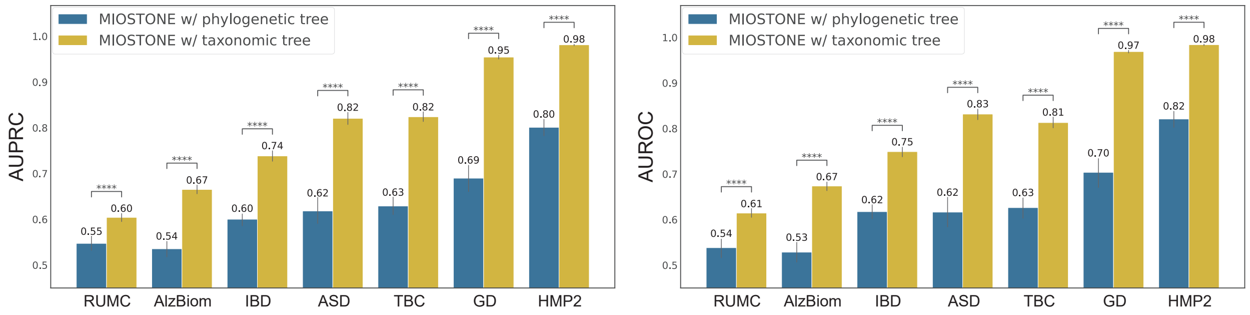

Figure S13: **MIOSTONE demonstrates superior performance in using taxonomy-encoding architectures.** While MIOSTONE can emulate any hierarchical correlation among taxa within its architecture, alternatives, such as phylogenic trees, perform significantly worse than the taxonomy-encoding architectures.

References

T. Chen and C. Guestrin. XGBoost: A scalable tree boosting system. In ACM SIGKDD International Conference on Knowledge Discovery and Data Mining, KDD '16, pages 785–794, New York, NY, USA, 2016. ACM.

D. Fioravanti, Y. Giarratano, V. Maggio, C. Agostinelli, M. Chierici, G. Jurman, and C. Furlanello. Phylogenetic convolutional neural networks in metagenomics. BMC Bioinformatics, 19:1–13, 2018.

C. G. Gonzalez, R. H. Mills, Q. Zhu, C. Sauceda, R. Knight, P. S. Dulai, and D. J. Gonzalez. Location-specific signatures of Crohn's disease at a multi-omics scale. Microbiome, 10(1):133, 2022.

M. He, N. Zhao, and G. A. Satten. MIDASim: a fast and simple simulator for realistic microbiome data. Microbiome, 12(1):135, 2024.

F. Pedregosa, G. Varoquaux, A. Gramfort, V. Michel, B. Thirion, O. Grisel, M. Blondel, P. Prettenhofer, R. Weiss, V. Dubourg, J. Vanderplas, A. Passos, D. Cournapeau, M. Brucher, M. Perrot, and E. Duchesnay. Scikit-learn: Machine learning in Python. Journal of Machine Learning Research, 12:2825–2830, 2011.

D. Reiman, A. A. Metwally, J. Sun, and Y. Dai. PopPhy-CNN: a phylogenetic tree embedded architecture for convolutional neural networks to predict host phenotype from metagenomic data. IEEE Journal of Biomedical and Health Informatics, 24(10):2993–3001, 2020.

D. Sharma, A. D. Paterson, and W. Xu. TaxoNN: ensemble of neural networks on stratified microbiome data for disease prediction. Bioinformatics, 36(17):4544–4550, 2020.

Y. Wang, T. Bhattacharya, Y. Jiang, X. Qin, Y. Wang, Y. Liu, A. J. Saykin, and L. Chen. A novel deep learning method for predictive modeling of microbiome data. Briefings in Bioinformatics, 22(3):bbaa073, 2021.

J. Zhai, Y. Choi, X. Yang, Y. Chen, K. Knox, H. T. III, J.-H. Won, H. Zhou, and J. J. Zhou. DeepBiome: a phylogenetic tree informed deep neural network for microbiome data analysis. Statistics in Biosciences, pages 1–25, 2024.

Table S1: The details of the real datasets investigated by MIOSTONE.

Dataset	Sample size	Feature size	Data source	Notes
AlzBiom	175 samples (75 amyloid-positive and 100 healthy control)	8,350 taxa	EBI-ENA ID: PRJEB47976	The sequencing data is clean (QC'ed, host-filtered).
ASD	60 samples (30 typically developing and 30 constipated ASD)	7,287 taxa	EBI-ENA ID: PRJNA451479	The sequencing data is raw (non-QC'ed).
GD	162 samples (100 Graves' disease and 62 healthy control)	8,487 taxa	EBI-ENA ID: PRJNA602729, PRJNA602731, PRJNA602732, PRJNA638403, PRJNA638404, PRJNA638405	Most samples have a pair of FASTQ files. However, 4 samples (three.lst) have a third, unpaired FASTQ file that is very small, and it should be excluded from the analysis. 12 samples have only one FASTQ file, which appears to be single-end sequences. Two samples: GA61 (SRR12000211) and GA89 (SRR12005695) are missing from the metadata. Therefore they were dropped from the data.
RUMC	114 samples (42 Parkinson's disease and 72 healthy control)	7,256 taxa	Qiita ID: 12975	20 samples in BIOM are missing in metadata. These samples were dropped.
TBC	113 samples (46 Parkinson's disease and 67 healthy control)	6,227 taxa	Qiita ID: 14476	5 samples in BIOM are missing in metadata. These samples were dropped.
IBD	174 samples (108 Crohn's disease and 66 ulcerative colitis)	5,287 taxa	Qiita ID: 12675	The dataset contains metagenomic sequencing data and associated metadata. More details can be found at: `https://qiita.ucsd.edu/study/description/12675`
HMP2	1,158 samples (728 Crohn's disease and 430 ulcerative colitis)	10,614 taxa	Qiita ID: 11484	The dataset contains metagenomic sequencing data and associated metadata from the Human Microbiome Project. More details can be found at: `https://hmpdacc.org/ihmp`
